# Role of Iron Oxide (Fe_2_O_3_) Nanocomposites in Advanced Biomedical Applications: A State-of-the-Art Review

**DOI:** 10.3390/nano12213873

**Published:** 2022-11-02

**Authors:** Mehrab Pourmadadi, Erfan Rahmani, Amin Shamsabadipour, Shima Mahtabian, Mohammadjavad Ahmadi, Abbas Rahdar, Ana M. Díez-Pascual

**Affiliations:** 1 School of Chemical Engineering, College of Engineering, University of Tehran, Tehran 14174, Iran; 2 Department of Materials Engineering, Shahreza Bramch, Islamic Azad University, Shahreza, Isfahan 61349-37333, Iran; 3 Department of Physics, Faculty of Sciences, University of Zabol, Zabol 538-98615, Iran; 4 Universidad de Alcalá, Facultad de Ciencias, Departamento de Química Analítica, Química Física e Ingeniería Química, Ctra. Madrid-Barcelona, Km. 33.6, 28805 Alcalá de Henares, Madrid, Spain

**Keywords:** iron oxide nanoparticles, nanomaterials, nanotreatment, nanocarrier, drug delivery, tissue engineering, wound dressing

## Abstract

Nanomaterials have demonstrated a wide range of applications and recently, novel biomedical studies are devoted to improving the functionality and effectivity of traditional and unmodified systems, either drug carriers and common scaffolds for tissue engineering or advanced hydrogels for wound healing purposes. In this regard, metal oxide nanoparticles show great potential as versatile tools in biomedical science. In particular, iron oxide nanoparticles with different shape and sizes hold outstanding physiochemical characteristics, such as high specific area and porous structure that make them idoneous nanomaterials to be used in diverse aspects of medicine and biological systems. Moreover, due to the high thermal stability and mechanical strength of Fe_2_O_3_, they have been combined with several polymers and employed for various nano-treatments for specific human diseases. This review is focused on summarizing the applications of Fe_2_O_3_-based nanocomposites in the biomedical field, including nanocarriers for drug delivery, tissue engineering, and wound healing. Additionally, their structure, magnetic properties, biocompatibility, and toxicity will be discussed.

## 1. Introduction

Transferring from the macro-sized world to nano-sized materials seems to change the world immensely, and the fast-growing advances in nanotechnology have led to excellent clinical achievements [1]. Nanotechnology offers tremendous applications in biomedical engineering for loading therapeutic cures, specifically in cancer treatments. Thanks to their unique surface features, nanoparticles (NPs) can offer a promising platform for loading therapeutic agents in drug delivery systems [2,3,4,5,6,7,8]. The interaction of NPs with biological systems has offered the simultaneous possibility of targeted drug delivery, monitoring, and therapy. Anti-cancer loaded Nanocarriers could escape from the recognition of the reticuloendothelial system (RES), increasing circulation time makes them more preferable than the traditional cancer therapies, such as chemotherapy, by enhancing drug accumulation at tumor sites to reduce systemic toxicity. Free anti-cancer drugs damage health cells which cause harmful side effects [9,10]. Additionally, nanomaterial-based carriers improve the oral bioavailability and solubility of the chemotherapeutic agents and lessen the required administrated doses of drugs [11,12,13,14]. Another merit of nano delivery systems is their ability to decrease drug resistance, which could be employed to treat resistant cancers by utilizing co-delivery systems [15]. Hence, nanocarriers could be used in drug delivery systems to load the therapeutic agent, protect it from physicochemical degradation, target ligands because of their functional groups, enter the cells, and release the anti-cancer agent with high therapeutic efficiency.

In contrast with other inorganic NPs (e.g., gold and carbon-based NPs), magnetic NPs can be decomposed to Fe, especially in acidic cell compartments (such as lysosomes), declining the potential long-term toxicity of NPs [16]. Additionally, iron oxide nanoparticles (IONPs) can be coated in a core-shell structure to prevent their agglomeration as a cluster. In this way, they could be gradually eliminated from the bloodstream [17]. The surface coating also improves the vascular permeability of the drug and facilitates its transport across the endothelial barrier [18]. It can also attach to particular aptamers or antibodies to target specific cells. The modified surfaces could offer diverse targeted moieties such as peptides, antibodies, and small molecules that can be conveniently coated on magnetic nanoparticles’ surfaces thanks to the intrinsic magnetic features [19,20].

Iron oxide has unique properties that make it an attractive nanoparticle in biomedical applications [21,22,23]. Especially, Fe_2_O_3_ nanoparticles can enhance the stability and permeability of therapeutic agents through tissues, bringing about a high circulation time [24]. Consequently, applying Fe_2_O_3_-based nanocarriers presents an efficient treatment with less drug dosage requirement. Additionally, according to their capacity for loading drugs, they would provide controlled release of drugs, which minimizes toxicity and eliminates overdosage by increasing the accumulation of the medicine in non-healthy sites [25]. Hence, these advantages, alongside the biocompatibility of Fe_2_O_3_ NPs, have attracted a significant amount of attention among the researchers during past few decades. Characteristics of these NPs depend on their morphology and crystal structure, size, and preparation route [26].

As a transition metal oxide, iron oxide possesses a variety of stoichiometric and crystalline structures, such as magnetite (Fe_3_O_4_), maghemite (γ-Fe_2_O_3_), Hematite (α-Fe_2_O_3_), Barite, (β-Fe_2_O_3_) maghemite, and (ε-Fe_2_O_3_) magnetite wüstite (FeO) [27]. Hematite (α-Fe_2_O_3_) has shown the highest stability at ambient conditions due to its small bandgap (2.0–2.2 eV). A hematite crystal consists of iron atoms surrounded by six oxygen atoms (hexagonal). Two-thirds of the hexagonal interstices in α-Fe_2_O_3_ are occupied by Fe^3+^ cations in the (001) basal planes, leaving the tetrahedral sites unoccupied, while oxide ions (O^2−^) reside along the (001) plane of a hexagonal closed-packed lattice. This cationic arrangement would lead to the formation of pairs of FeO_6_ octahedrons, whose edges are shared by three neighboring octahedrons of the same plane and one face with an octahedron in an adjacent plane along the (001) direction [28]. With C3v symmetry, Hematite exhibits two Fe-O bond lengths. Tauc plot reveals the indirect bandgap for the α-Fe_2_O_3_ involving d-d transitions and a direct transition from O (2p) to Fe (3d) occurring for Eg > 3.2 eV [29]. Hematite is an environmentally friendly n-type semiconductor (Eg = 2.1 eV) with diverse applications in lithium-ion batteries, water treatment, gas sensors, photocatalysis, and water splitting for H_2_ generation.

The fundamental of targeted delivery of anti-cancer agents is enhancing permeability and retention (EPR), and one of its techniques is taking advantage of the physiological differences between normal and tumor cells. For example, cancerous sites have lower pH (5.4) in comparison with healthy ones’ (pH 7.4). Facilitating pH-responsive systems is an amazing way to enhance the accumulation of drugs in tumors. Surface modifications of Iron oxide nanoparticles, mainly because of their high specific area and porosity, make them a great candidate for being used as pH-sensitive vehicles in drug delivery systems (DDSs) [30]. Furthermore, the magnetic properties of iron oxide NPs can be applied as a novel route for delivering drugs and optimizing their dispensation through an exterior magnetic field to guide iron oxide-based nanocarriers to deliver drugs into a specific location in the body. Magnetic guidance can also boost the drug release at the tumor site [31].

In addition, magnetic nanoparticles have electrocatalytic features that give rise to rapid readout in redox-responsive delivery systems [32]. Controlled disassembly of redox-responsive drug delivery system results in rapid release of encapsulated medicines. Nanocarriers can be functionalized by enzyme-labile connections to achieve an on-demand enzyme-sensitive drug release system to decrement the adverse effects of therapeutic agents. An efficient enzyme-sensitive nanosystem can penetrate tumor tissues with odd features such as leaky vasculature and various enzyme expression profiles. Relevant enzymes can be employed to prepare Fe_2_O_3_ NPs with promising features. Due to their endogenous nature, enzymes exhibit superior selectivity and catalytic features in various biological processes. Therefore, they have been widely explored as target agents in diverse endogenous and exogenous stimuli-sensitive systems [33]. Moreover, enzyme sensitivity increases both the rate of drug release into the tumor and the amount of drug that accumulates within it. Thus, redox responsive dual-stimuli responsive DDSs can enhance anti-cancer therapy by raising drug accumulation, promoting tumor targeting, and accelerating drug release. Further stimuli can be incorporated into redox reactive delivery systems to control release and minimize the side effects effectively [34].

In addition, to enhance mechanical and biological performances, nanoparticles, because of their small size that helps them to simulate extracellular matrix components of tissues, have been used recently in tissue engineering as substitutes for damaged and diseased tissues [35].

Specifically, different structures of iron oxide nanocomposites have a high surface-to-volume ratio, which has increased their application in this field of biomedical engineering. This feature can increase drug loading and entrapment efficiency and can cause a gradual drug release pattern [36,37]. It should be noticed that the extended surface area is produced in iron oxide nanocomposites due to the addition of iron oxide nanoparticles to the prepared hydrogels, in other words, by adding iron oxide nanoparticles to the fabricated hydrogel, these nanoparticles introduce porosity to the system and the final iron oxide nanocomposites would be a porous platform with a considerable ratio of surface area to volume. The BET assessment that has been carried out by Nurul Hidayah et al. [38], revealed a significant increase in the specific surface area of reduced graphene oxide from 25 m^2^/g to 51 m^2^/g after incorporation with iron oxide nanoparticles.

For example, the physical and chemical properties of Fe_2_O_3_ nanoparticles can apply growth factors are applied to scaffolds to induce bone-forming cell regeneration that would overcome the difficulties of producing complicated organs such as bones [39,40]. Furthermore, for tissue engineering applications, scaffolds needed to be porous for better mass transportation of O_2_, CO_2_, and other nutrients with extended surface area [41,42]. These requirements can be perfectly fulfilled by employing iron oxide nanoparticles in the structural fabrication of the scaffolds.

In addition, iron oxide nanoparticles benefit shapes and non-toxic nature that make them applicable for wound healing, too. They could be used in wound dressing materials to stimulate the movements through the different healing phases in acute and chronic wounds [43]. Moreover, some characteristics of IONPs including spacious surface, high specific area, and porous structure give the Fe_2_O_3_-based nanocomposites and hydrogel higher absorption capacity, water, and oxygen permeability [44,45,46]. These special features of IONPs can introduce an applicable wound dressing for biomedical applications.

Potential of Fe_2_O_3_ for being functionalized and coated by suitable polymers for making these NPs non-toxic, super magnetic, biocompatible, and biodegradable, introducing them as the best materials for biomedical applications including drug delivery (magnetic-sensitive), tissue engineering (bone tissue regeneration), and wound dressing. In this study, different structures and specific characteristics of IONPs were examined, and previous investigations on their advanced biomedical applications were reviewed.

## 2. Structure of Iron oxide Nano Particles

**Figure 1:**
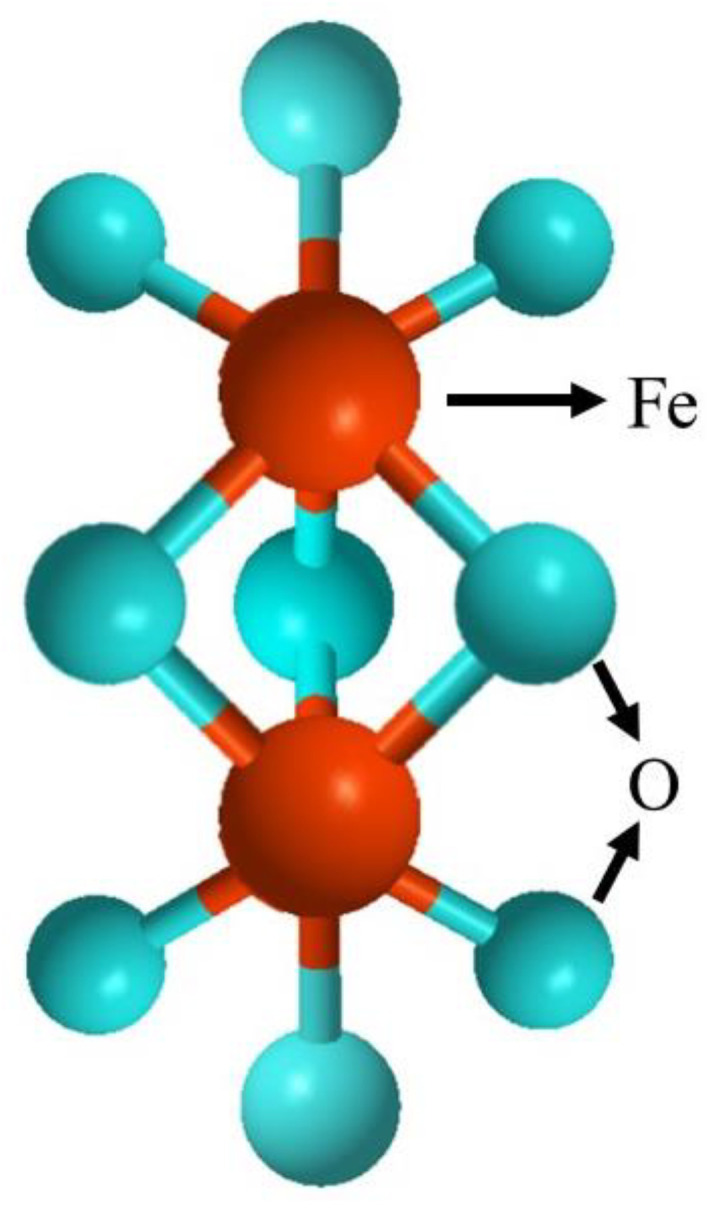
Structure of α-Fe_2_O_3_ (Hematite).

Fe_2_O_3_ and Fe_2_O_3_-based materials with diverse sizes and shapes (NPs, nanotubes, nanorods, spindles, hollow, and porous nanostructures) were prepared by hydrothermal, chemical precipitation, sol-gel, anodization, and thermal decomposition approaches. The hydrothermal method is desirable as it offers efficient control of size, morphology, and agglomeration while lower impurities enter the hydrolyzed product. It also benefits from relatively low reaction temperature, cost-effectiveness, and environmental compatibility. Moreover, Fe_2_O_3_ possesses two interchangeable crystalline forms (α-Fe_2_O_3_ and γ-Fe_2_O_3_). The structure of α-Fe_2_O_3_ is represented in Figure 1. At higher temperatures, γ-Fe_2_O_3_ (maghemite) can be transformed to α-Fe_2_O_3_ (Hematite) as α-Fe_2_O_3_ is the most thermodynamically stable phase. The transition from α-Fe_2_O_3_ to γ-Fe_2_O_3_ requires a nitrogen atmosphere with adequate temperature control or the application of a reducing agent. The morphology generally remains unchanged by the transition between α-Fe_2_O_3_ and γ-Fe_2_O_3_, which could be an applicable approach to provide the controllable preparation of Fe_2_O_3_ with a modulated crystalline structure. There are a variety of magnetic phenomena in the α-Fe_2_O_3_, which is due to the exchange bias and memory effects. Furthermore, in small Fe_2_O_3_ nanoparticles (smaller than 10 nm), superparamagnetic behavior and spin-glass properties are observed [47]. These hexagonal-structured nanoparticles are chemically active, avoid clearance by immune cells, and have a noticeable retention time, electrical conductivity, and biocompatible nature, all of which make them good candidates for biomedical applications [48,49].

## 3. Drug Delivery Application of Fe_2_O_3_-Based Nanocomposites

In biomedical settings, pH-responsive systems may benefit from inducing physiochemical alterations due to the wide range of pH values seen in the human body [27,50,51]. Gastric passage elevates pH in oral medication delivery systems, including insulin administration, causing acrylic-based polymers to expand and discharge the medicine [52]. A negatively charged surface would be formed in pH 7.4 settings, causing the nanoparticle to discharge the loaded medicine. This is another advantage of functionalized nanoparticles with positively charged surfaces that trigger anionic drug loading at lower pH [53]. By covering Eudragit-S100 polymer using ibuprofen-loaded Fe_2_O_3_ magnetic mesoporous silica nanocomposites tablets, Xing et al. [54] create a dual-stimulus-responsive platform. Nanoparticles with tailored releasing capability were created by the magnetic characteristics of Fe_2_O_3_ when used in conjunction with an externally applied magnetic field. More medication was delivered into modeled proximal intestinal fluid when the pH-sensitive polymer was used, as opposed to modeled stomach fluid.

pH-responsive mechanisms may also be used at the cellular level. DNA and other therapeutic compounds are damaged when the pH of early endosomes, sorting endosomes, and multivesicular bodies drops rapidly following endocytosis [55]. Injury caused by intracellular distribution may be avoided using polymers that safeguard endosomal compartments. A group led by Alexander et al. [56] using γ-Fe_2_O_3_@polymerized 2-(dimethyl amino) ethyl methacrylate developed dual-responsive core-shell nanoparticles for plasmid DNA delivery throughout CHO-K1 cells. Researchers found that, compared to polyethyleneimine, magnetic core-shell nanoparticles might experience bidirectional pH-dependent temperature-induced aggregation and enhanced efficacy of gene transport with no extra cytotoxic effects. Chemotherapy, which is the typical cancer treatment strategy, can assault both diseased and normal cells due to the application of nonspecific targeting medicines [57]. By increasing the specificity of drug-loaded NPs and ameliorating permeability and retention (EPR) events, intelligent pH-responsive platforms can help reduce complications [58,59].

**Figure 2:**
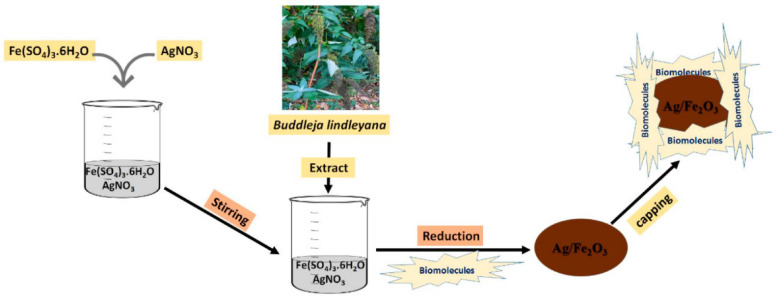
The reduction procedures of the Ag/Fe_2_O_3_ nanocarrier for loading biomolecules [66].

Tumors develop rapidly, accumulating lactic acid and lowering the pH of these areas (pH 4–6) in comparison to physiological status (pH 7.4) [50,60]. The highly expanded surface area, appropriate porosity, and capacity to be coated by polymers as core-shell nanoparticles make Fe_2_O_3_ NPs such an ideal carrier for pH-responsive drug delivery platforms, moreover, there is a potential for the reduction of Fe_2_O_3_ based nanocarriers for loading biomolecules (Figure 2) [61,62,63,64,65,66]. These characteristics provide sufficient room for loading antitumor medications. Regarding the delivery of doxorubicin, Sheng et al. [67] created polyethylene glycol-functionalized γ-Fe_2_O_3_ nanoparticles, then tested for pH influences on drug distribution and alternating magnetic field treatment. They observed that total discharges were 32 and 63% at pH levels of 7.2 and 5.5, respectively. Daunorubicin hydrochloride was loaded into a γ-Fe_2_O_3_/ZnO nanocomposite by Maiti et al. [63], and then drug delivery was measured at pH 5.5 and 7.5. When used as a model for tumor locations, the acidic setting aided in the drug delivery. To make things even better for medication distribution, mesoporous ZnO can load large amounts of medicines. With curcumin anticancer medication, Patil and colleagues created functionalized chitosan-coated γ-Fe_2_O_3_ nanomaterials activated by a change in pH [68]. They found that the discharge frequency at pH 6.0 was approximately 20% higher than at pH 7.4, suggesting the nanocarrier’s capacity for cancer treatment. Various proportions of oxidized pectin/chitosan were loaded in nano γ-Fe_2_O_3_ to improve the antitumor properties of the 5-FU medication by Li et al. [69] pH levels below 7 and temperatures greater than 36.5 °C demonstrated the system’s pH and thermo-sensitivity by showing a larger swelling frequency. The MMT test against L929 and MCF-7 cell cultures was used to determine the synthesized composite’s biocompatibility and cancer-killing abilities. Due to this, 5-FU may be more effectively targeted, and its anticancer capabilities are enhanced when the oxidized pectin/chitosan/γ-Fe_2_O_3_ NPs are used.

The exceptional electrochemical redox capabilities of magnetic nanoparticles may be attributed to their substantial electrocatalytic characteristics in a single structure and their composites. It may be formed by optimizing factors, including the transmission of ions by solid NPs because of their high penetration, which accelerates internal dispersion’s phase changes [32]. With the help of their redox capabilities, magnetic nanomaterials can gather and concentrate the desired electrochemical data for quick reading [70].

The most often employed NPs in this sector are γ-Fe_2_O_3_ (oxidized state, Fe^3+^) and Fe_3_O_4_ (two oxidized states, Fe^2+^ and Fe^3+^) in MNMs-based composites, respectively [71,72]. Magnetic nanoparticles and targets discovered by cyclic voltammetry influence the electrodes’ predicted redox potential [73].

Poly (ethylene glycol) and poly(ε-caprolactone) disulfide bonds (PEG SS PCL) were used to create biodegradable reduction-responsive micelles for the administration of super-paramagnetic iron oxide (SP IO) nanoparticles and doxorubicin (a chemotherapeutic drug). In the main chain of disulfide bonds, the amphiphilic deblock copolymer has redox reaction characteristics. Doxorubicin (DOX) was loaded 32% throughout magnetic nanomicelles. Consolidation of self-assembled PEG-PCL micelles with oleic acid and water was achieved using coarse-grained molecular dynamics (CG-MD) modeling. In comparison, the hydrophobic and hydrophilic proportions of each copolymer block were identical, and each oleic acid was similarly attached to magnetic nanomaterials [74].

To modulate camptothecin pharmaceutical discharge from the mesoporous silica pathways and achieve a dynamic dual-mode MRI contrast, researchers employed superparamagnetic iron oxide nanoparticles (SPION) coated by acid, oxidation stress, and redox-sensitive manganese oxide (MnOx). The efficiency of responsive therapy has been tested throughout pancreatic tumor cells and tumor-bearing animals, and results have supported the performance of Non-vehicle MnOx-SPION@MSn@CPT fighting cells [75]. To further improve biodistribution and efficacy for convection-enhanced delivery (CED) of BG to GBM, iron oxide superparamagnetic nanomaterials were developed. The nanomaterials (NPCP-BG-CTX) consist of a magnetic core surrounded by a surface coating of redox-responsive chitosan-PEG copolymer altered by BG covalent attachment and peptide chlorotoxin tumor (CTX). NPAC-BG-CTX demonstrated precise in vitro BG transport in human GBM cells under decreased intracellular circumstances, allowing for controlled and targeted BG discharge. MGMT activity and TMZ toxicity were significantly reduced in cells administered with NPCP-BG-CTX. Animal studies in mice with primary human GBM xenografts showed that CED from NPCP-BGCTX had good in vivo distribution volume (Vd). When NPCP-BG-CTX and NPCP-CTX were used in conjunction, the mean survival rate in both medicated and unprotected animals increased by three times [76].

Enzymes are widely used by internal stimulus-responsive nanocarriers since they are involved in a wide range of biochemical and physiological mechanisms. Enzyme transcription at specific places is critical in a variety of pathological processes. In addition to indicating a sick condition, it may also be modified to transport medications with more safety to the appropriate region and serves as a possible screening tool [33]. In contrast to other stimuli, enzymatic processes are more powerful, efficient, and accurate, providing extra benefits by intracellularly participating in various metabolic reactions. The transcription of enzymes, including proteases, phospholipases, hydrolases, and glucosidase, increases in a variety of malignancies that use growth factors to increase cell populations. These specific enzymes detect and split intelligent nanocarriers comprised of substances such as Fe_2_O_3_ to deliver the payload, namely the medicine, to the targeted place. Multifunctioning is typically supported by adding more than one molecule to the surface of a material [77]. The overexpression of MMP-14 in MMTV-PyMT cells was shown to be much more hazardous than the downregulation of MMP-14 throughout fibroblasts. CLIO-ICT (a platform for iron oxide nanocarrier) was produced by Ansari et al. [78], to be used as theragnostic nanomaterials for magnetic resonance imaging (MRI) and medication administration. CLIO-ICT was created by linking an azademethylcolchicine (ICT) conjugator to an MMP-14-cleavable region. MMP-14 overexpression caused considerable toxicity among MMTV-PyMT cells, as seen by comparison to fibroblasts with modest frequencies of MMP-14 transcription.

Furthermore, in MR imaging of MMTV-PyMT tumor-bearing mice given by CLIO-ICT, preferential accumulation of nanomaterials in the cancerous site was detected. An enzyme-response, multifunctional DOX-SMNP combination was produced employing clicking chemical properties. Due to the upregulation of a specific enzyme in these live cells, the chemotherapeutic drugs released by this SMNPs-combined medication are selectively controlled. For the detection of intracellular drug delivery and tumor cell scanning, this drug-loaded nanoparticle combination demonstrates a potential mixture of fluorescence and magnetic resonance imaging methods performed in real time. Prospective uses of the multipurpose drug-coated nanostructures include tumor-targeted medication delivery and concurrent diagnostic or detection of therapeutic effects [79].

## 4. Fe_2_O_3_ in Magnetic-Responsive Drug Delivery Systems

Site-specific drug distribution may be achieved by directing IONs caused by a confined external magnetic field, which makes use of the magnetic characteristics of iron oxide nanomaterials. Several illnesses, including tumors and inflammation, have been shown to benefit from this strategy [80]. To govern the nanomaterials’ passage through the circulation and their concentration at the intended places, the magnetic reaction of IONs is highly associated with their physicochemical qualities; more precisely, the saturation magnetization of the manufactured nanostructures should be significant [81]. Some scientific teams are creating magnetic nanocarriers with unique structural properties that have examined this site-directed function. There was a publication in 2012 by the group of Wagstaff et al. [82] about how to make gold-covered iron oxide nanomaterials loaded with a platinum-containing chemotherapeutic agent (cisplatin). Coprecipitation and oxidation of iron oxide nanocrystals resulted in the formation of maghemite during this investigation. A technique known as “incremental hydroxylamine seeding” was used to cover the nanomaterials with gold [83]. Subsequently, thiolation was used to coat the particles. Earlier, the same group discovered substances such as thiolated polyethylene glycol (PEG) linkers [84,85]. Eventually, robust coordination linkages involving the PEG linker were used to load cisplatin onto the magnetic nanocarriers. Using a naked magnet, the nanocarriers were tested in vitro and shown to have a 110-fold enhancement in cytotoxic effects on human ovarian cancer cell lines A2780, as well as particular cell development suppression.

Unterweger et al. [86] created IONs coated with dextran/hyaluronic acid, which have cisplatin-carrying IONs66. The loading of medications, including cisplatin, and the targeting of overexpressed CD44 receptors throughout malignant cells, were made possible by applying hyaluronic acid (HA) [87]. Following the dextran-coated IONs amination, they were covered by lower-molar mass HA (acquired via enzymatic breakdown of HA) [88]. After forming a polymer-metal combination with HA, cisplatin was successfully incorporated into the resultant nanocarriers, which were noted for their effective drug entrapment (43.2 ± 0.2%). A beginning 30-minute explosive discharge was accompanied by an ongoing discharge for 48 h, which was described by the researchers as being typical for surface-bound drugs in their study. A substantial enhancement in drug delivery has also been evidenced in the availability of hyaluronidases, which are extensively discovered in tumors and make these nanostructures especially encouraging for tumor-targeted drug delivery [89]. This is made even more promising by their regulation through magnetic power illustrated by the uptake of nanomaterials containing a neodymium magnet. PVA-coated iron oxide nanostructures loaded with doxorubicin have also been explored by Nadeem et al. [90], who investigated the magnetic manipulation of IONs for specific drug delivery purposes. These nanocarriers exhibited good management for magnetically directed medication delivery by providing a magnetic flux. The antineoplastic medication mitoxantrone was adsorbed on the HSA shell of iron oxide nanostructures (average inorganic diameter of around 7 nm) synthesized by Zaloga et al. [91] using lauric acid (LA) and HSA coatings. Over 72 h, such nanocarriers showed improved durability and linear discharge pharmacokinetics. Using an in vitro magneto-guided approach, researchers were able to convincingly demonstrate the site-specific curative impact of these nanocarriers [92].

In a recent study, Jeon et al. [93] demonstrated the synthesis of polymerized β-cyclodextrin covered IONs that, via host-guest association, were capable of loading polymerized paclitaxel (a chemotherapeutic medication renowned for its limited solubility in water). Due to the strong magnetism of the produced nano assemblies, they may be used as magnetically directed drug delivery platforms. The improved antitumor efficacy of these platforms was shown in vivo throughout CT26-bearing mice due to magnetically-mediated targeting.

**Figure 3:**
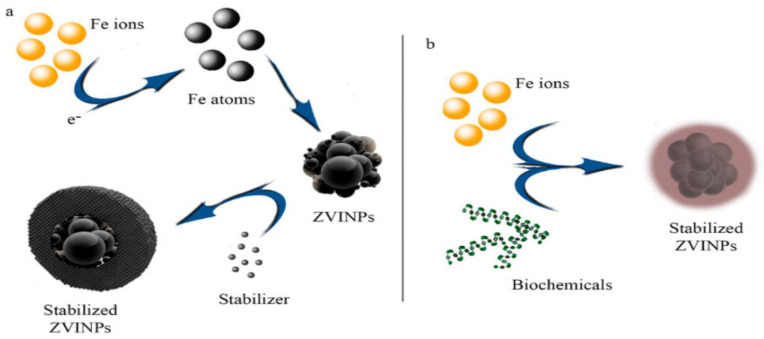
Chemical (**a**) and biological (**b**) reduction of iron ions to zero-valent iron nanoparticles (ZVINPs) and their stabilization [104].

Normal cells suffer adverse effects when the body’s temperature is elevated during tumor hyperthermia therapy. An external magnetic field would allow for targeted hyperthermia, which reduces the damage to healthy tissues and reduces the needed medication dosage [94,95]. External signals may act as a guide for transporting the medicine to the tumor location and initiating pharmaceutical discharge [96]. Since malignant cells are less resistant to temperature rise than healthy ones, utilizing MNPs in the carrier generates a thermal flow that aids in the destruction of diseased cells [97,98]. An external source’s proximity to the targeted region influences the magnetic-responsive drug delivery platform’s precision; as tissue profundity increases, the required intensity magnetic flux decreases [99]. As a consequence, this technique is confined to surface organs and so cannot be used to treat pulmonary or hepatic tumors [100]. Aside from that, frequencies greater than 8–16 kA.m^−1^ generate eddy currents that are damaging to normal organs [101,102]. Owing to their super magnetic capabilities, biocompatibility, high specialized region, appropriate nano-sized particulates, and lower toxic effects, Fe_2_O_3_ nanomaterials are one of the most promising alternatives for magnetic-responsive devices according to the researchers [31,103,104] (Figure 3). Improved drug loading potential, increased carrier durability, and opportunity for future functionalization might be achieved by coating iron oxide NPs using appropriate polymers [67,105,106,107,108]. In vitro experiments using human hepatocarcinoma SMMC-7721 cells, Yan et al. [109] employed a combination of Fe_2_O_3_ nanomaterials and magnetic fluid hyperthermia (MFH) and found that apoptosis was dose-dependent, and proliferation was inhibited. Due to their absorption capabilities, Fe_2_O_3_ nanomaterials have magnetic sensitivity, which causes the magnetic fluid temperature to rise by around 7 °C. An AC magnetic field was used to study the emission of heat from the nanohydroxyapatite matrix-encased γ-Fe_2_O_3_ NPs. Investigations on the cytotoxic effects of human (sarcoma osteogenic) SAOS-2 cell lines in vitro demonstrated minimal toxicity, indicating that they may be employed in magnetic hyperthermia [110].

Due to low viral concentration in the targeted tissue, gene therapy faces a key drawback: delayed vector deposition [111]. The specificity can be improved by attaching targeted ligands to viral particles; however, this is insufficient [112]. The difficulty may be solved by introducing an exterior alternating current magnetic field to gene vectors. For this technique of gene delivery, Scherer et al. [113] attached gene vectors to superparamagnetic nanoparticles. Transduction effectiveness was shown to be great in vivo, and the delivery time of genes was lowered to minutes, supporting the idea that magnetic field guidance may be used to overcome fundamental obstacles. Fe_2_O_3_ uses in magnetic-responsive biological devices are listed in Table 1.

## 5. Biocompatibility and Toxicity of Fe_2_O_3_-Related Drug Carriers

The immunological system reaction after delivery and the inherent toxicity of the carrier and/or biodegradation of metabolites are all variables that impact the biocompatibility of a drug nanocarrier. Due to alterations in cell/tissue bioavailability and clearance/metabolization related to the nanocarrier, the toxic effects pattern of the medication may be raised or lowered. In physiological enclaves where the medication cannot disperse on its own, aggregation may develop.

After drug delivery, there must be a rapid removal of drug-related colloids and their breakdown products to minimize toxicological effects. Dosage, chemical constitution, size, shape, solubility, surface chemistry, mode of prescription, biodegradability, pharmacokinetics, and bioavailability are a variety of parameters affecting magnetic particle toxicity [119].

**Figure 4:**
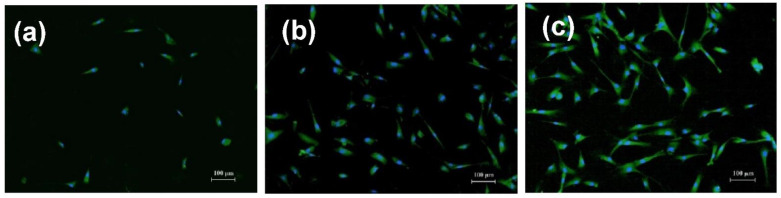
Fibroblasts were cultured on the surface of gel-based substrate (**a**), with 1.2% Al_2_O_3_ (**b**), and with 1.2% Fe_2_O_3_ NPs (**c**). Cell nuclei and the cytoplasm were stained with DAPI and pyrazolone yellow, respectively [122].

The structure of the NP surface has a major role in the NP–cell association, as previously stated. The effectiveness of IONP cell absorption and internalization is a precondition for enhanced medication administration, enhanced diagnostic efficiency, and other usages of IONPs. When nanoparticles are incubated in a cell culture environment, it is possible that they could change the pattern of cell attachment, which will have consequences for the cell’s shape and cytoskeleton and its multiplication, differentiation, motility, and longevity (Figure 4) [120,121,122].

When exposed to physiological circumstances, bare Fe_2_O_3_ nanocrystals create damaging free radicals [123,124]. The cores of the magnets should be coated with additional particles to circumvent this limitation. In this method, not only would the colloidal durability of the nanocomposite improve, but the performance probability of the experiment would also rise. Throughout drug delivery platforms, low-toxicity coated iron oxides may have an EPR impact on certain cells [125]. Particle accumulation may also be prevented by using an appropriate covering [126].

Magnetic Fe_2_O_3_-based nanomaterials were manufactured by Dumitrache et al. [127] and contrasted to l-DOPA-coated nanostructures. Thermal conductance and distribution in biological medium (physiologic pH) were much enhanced following coating. Dopamine was used as a biocompatible surfactant in the research of Li et al. [128] to boost the durability of a nanocomposite with a nucleus of α-Fe_2_O_3_. α-Fe_2_O_3_@dopamine was administered to a mouse and was shown to be non-toxic to live cells when incubated with NIH3T3 cells. Furthermore, the PA indication of the mouse’s cerebral vascular system increased when the created theragnostic platform was administered, compared to commercialized contrast compounds. In vitro grown primary human fibroblasts (hTERT-Bj1) were used to test the biocompatibility of IONPs with and without a PEG surface coating [129]. Without PEG coatings, relative to the controls, the 50 nm diameter uncoated IONPs decreased cell adherence by 64%; however, a significant alteration was not seen with PEG-coated nanoparticles. Several cytoplasmic vacuoles with the disordered cytoskeleton and defective and disrupted cell membrane were seen throughout the NP-incubated cells without coating.

Even though that they exhibited a greater cell absorption relative to uncoated NPs, PEGylated MNPs, on the other hand, did not disrupt the fibroblast cytoskeleton. If PEG is present on the particle surface, the NP/cell interaction will be altered, and this might be due to variations in the kinetics of NP metabolic activities.

Comparable apoptosis and cytotoxicity were seen in hTERT-BJ1 primary human fibroblasts incubated with dextran-coated IONPs relative to those incubated with uncoated IONPs [130]. There was no difference in cell absorption between dextran-coated and uncoated NPs; however, the dextran-coated NPs were more likely to produce cellular mobility and membrane rupture. In contrast to dextran-coated and uncoated NPs, albumin-coated IONPs did not affect cell survival [131,132]. Since albumin interacts with membrane phospholipids/fatty acids, the albumin-coated NPs just marginally disrupted the membrane. Nonetheless, in comparison to the uncoated and dextran-coated nanoparticles, the toxicity of such NPs was much reduced totally. A remarkable finding was that the albumin-coated nanoparticles were internalized at a lesser rate compared to the uncoated and dextran-coated particulates. This raises the possibility that the NP toxicity outlined above may be influenced by the various amounts of intracellular particulate. In vitro studies have shown that various particle sizes may have diverse impacts on cells. For example, relative to 0.5-m-sized IONPs, 30-nm-sized types had much larger harmful impacts [133]. An effect on cellular destruction was detected depending on cell size and dosage whenever the A549 alveolar epithelial cells were incubated.

Some of the changes in cell culture caused by NP incubation may be responsible for the in vitro cytotoxic effects of MNPs M [134]. For example, the absorption of OH– ions onto the surface of naked (uncoated) iron oxides causes these materials to be negatively charged throughout the aqueous solution. Counterions may be attracted to the resultant electric field, which may aid in protein adhesion. Furthermore, the presence of Cl– ions throughout the cell culture media may potentially compete with the Fe, resulting in a change in the pH of the medium and the surficial properties of the nanoparticles. As a consequence of these connections with the cell culture medium, which result in modifications, including fluctuations in the ionic concentration and protein activity, cells may become detached from the medium and die as a result. MNPs coated with a surface layer, on the other hand, exhibit low interaction with their surrounding environment in the cell culture medium, which is most likely since there are fewer sites available on the particle surface for protein attachment [134].

**Figure 5:**
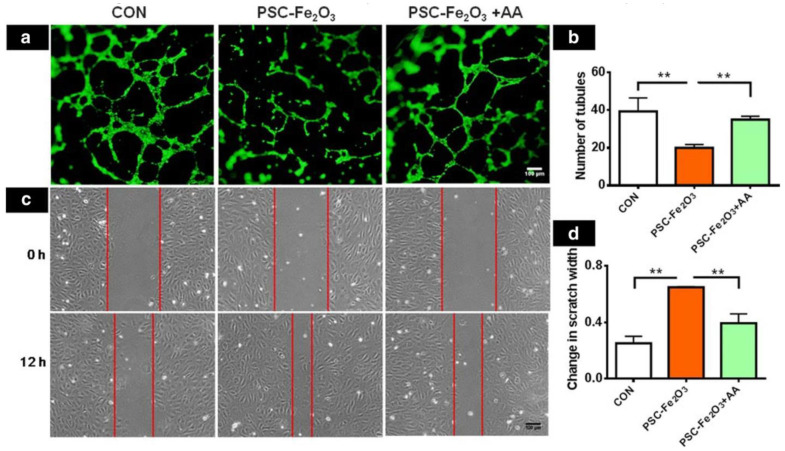
ROS scavenger AA conversed the function of endothelial cells happening EndMT. (**a**) AA conversed tubule production impairment of endothelial cell caused by PSC-Fe_2_O_3_. (**b**) measurement of endothelial meshes (*n*  =  3). (**c**) pictures of scratch morphology altering with time. (**d**) Relative scratch width alteration (*n*  =  2). Data are mean  ±  SD, one-way ANOVA with LSD-t, ** *p*  <  0.01 [139].

The form and behavior of cells may be influenced by the IONP surficial charge or other nanomaterials [135]. Endocytosis by multiple cell lines was boosted through the coating of 10-nm-sized IONPs containing DMSA (anionic charge) [136,137], which decreased cell adherence and survival of the PC12 pheochromocytoma cell line in a concentration-dependent manner [138]. PC12 spherical cells start to develop into neuronal cells following 48 h of treatment with nerve growth factor (NGF), resulting in mature neuritis that reaches the peripheral areas. The disruption of the cytoskeleton caused by anionic NPs, on the other hand, prevented neurite development and resulted in a significant reduction in the frequency of neurites formed per cell, as well as in the neuritis extent. Toxicities associated with these anionic MNPs may be depending on concentration, as seen by a reduction in GAP-43 protein production, which is linked to neuronal budding and neuronal functioning. Besides, Figure 5 represents the scratch morphology altering with time for a Fe_2_O_3_ based nanocomposite and shows the production impairment of endothelial cell caused by PSC-Fe_2_O_3_.

On the other hand, it has been shown that the anionic IONPs’ cytotoxicity varies depending on the kind of surface coating used. Cell lines from the SK-MEL-37 melanoma were cytotoxic to DMSA, citric acid, or lauric acid in distinct ways. DMSA-coated, citric acid-coated, and lauric acid-coated nanoparticles had IC50 (half-maximal inhibitory concentration) values of 2260, 433, and 254 g Fe/mL, correspondingly. Cellular death was seen when lauric acid-coated NPs were utilized at higher levels, including 800 g Fe/mL, as shown by morphological characteristics of the apoptosis profile, notably surficial blebbing, severe vacuolization, and chromatin contraction [140]. Therefore, even though the generated NPs have a comparable surface charge, the surface coatings may significantly modify their toxicity dose-dependently. Due to the variable nature of the intended use, it is necessary to conduct an individual cytotoxicity test for each sample.

Although early data on the security of MNPs may be revealed using in vitro toxicity assessments on different cell types; however, these tests may have restricted applicability since they do not match the real-life situations. Since under cell culture conditions, the NPs and their breakdown products (which might impair cell survival) stay in near the cells and may operate as a repository with a continual influence, the cytotoxic activity may be more severe in vitro compared to in vivo. On the contrary, if they are biodegradable, NPs are continually removed from the body [141]. Regarding pharmacological purposes, it is clear that the evaluation of in vivo security of MNPs is critical, as shown above.

**Figure 6:**
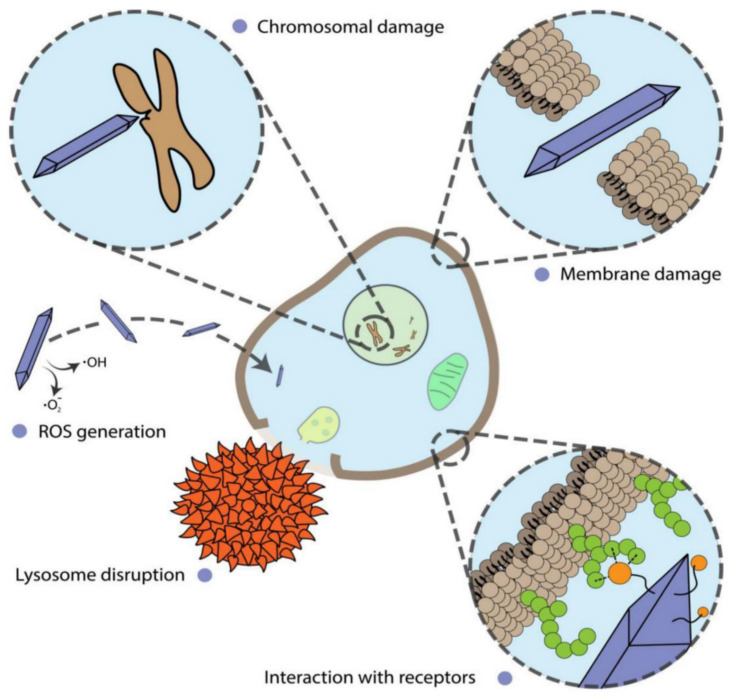
Various mechanisms of nanoparticles influence on cells [135].

Macrophages in the reticuloendothelial tissues remove intravenously administered IONPs from the systemic circulation, as previously stated and generally nanoparticles can induce diverse effects on cells (Figure 6) [142]. A lower pH, hydrolytic enzymes, and proteins involved in iron metabolic processes are among the features that allow these NPs to be picked by cells through receptor-mediated endocytosis and probably digested in the lysosomal compartment [143]. The liberated free iron is subsequently integrated into the body’s iron storage, where it is eventually discovered as hemoglobin and in part attached to transferrin before being removed mostly via the feces [144,145]. For an average adult male, the entire bodily iron storage is about 4000 mg. Small doses of iron infusion may not cause iron-related toxicity complications; however, larger concentrations may cause a rise in plasmatic iron levels due to the exceeded transferrin iron-binding capability. Oxidative stress and different toxicities, such as heart and liver damage, might be the result [146]. As a result, the cytotoxicity of MNPs has been investigated using a variety of delivery methods, including intravenous, intraperitoneal, and subcutaneous injection.

The security of IONPs stabilized using OA and Pluronic F-127 coatings after administration to rats at a dosage of 10 mg Fe/kg was studied [147]. Alanine aminotransferase, aspartate aminotransferase, and alkaline phosphatase concentrations in the blood did not change significantly or for a long period after the iron administration, although 55% of the administered iron had been deposited in the hepatocytes 6 h after the infusion. IONPs may produce oxidative harm to the phospholipidic cell membrane. Therefore, it was decided to assess the amounts of lipid hydroperoxide to determine the extent of the oxidative injury. The tissue-specific variations in lipid hydroperoxide concentrations were discovered. They were somewhat greater for the hepatic, splenic, and renal tissues compared to the other sites, including pulmonary, cerebral, and cardiac tissues. Nevertheless, even after one-week post injection, the elevated lipid hydroperoxide concentrations did not affect tissue shape or cellular stability, showing that the oxidative stress was modest and negligible at the administered dosage. MNP dosage and dosing duration are critical to ensuring the safety of this medication, as will be addressed more below.

To be sure, in several animal species, the MNPs exhibited an acceptable level of safety when supplied at various doses. As an example, rats given Ferumoxtran-10 at the 2.6 mg Fe/kg i.v. dosage was found to be protected, with no alterations in hemodynamic variables, whereas rats given 13 mg Fe/kg dosage had a gentle enhancement in aortic blood circulation; however, there were no arrhythmias or adjustments in the electrocardiogram, and no breathing toxicities were identified. Just very high single dosages of Ferumoxtran-10, including 126 mg Fe/kg and 400 mg Fe/kg, were treatment-related clinical symptoms in rats found to remain for 24 h post administration. There were modest alterations in body weight and hematological markers throughout rats after continuous intravenous treatment of 17.9 mg Fe/kg Ferumoxtran-10 (3–5 dosages). Ferumoxtran-10 was neither mutagenic nor hazardous to the maternal rats or rabbits given doses as high as 15 mg Fe/kg/day or repetitive administrations (up to < 50 mg Fe/kg/day throughout rabbits) [148]. Up to a dosage of 20 mg Fe/kg i.v. administration throughout dogs, Ferumoxtran-10 showed no treatment-related heart or kidney toxicities, but at elevated dosages, including 200 mg Fe/kg (75 times greater than the level employed in humans as an MRI contrast agent), modest cardiac toxic effects, hypotension, increased respiratory level, increased heart rate, as well as a significant rise in urinary discharge and salt excretion in the urine, were seen. Singular dosages of up to 25 mg Fe/kg throughout monkeys did not result in any significant evidence of cardiac toxicity or changes in blood parameters. Once treatment with iron infusion, blood iron concentrations were shown to be significantly elevated among rats, dogs, and monkeys; however, normal concentrations were restored following the therapy was stopped. It was shown that the dose-dependent toxicity of IONPs could be characterized whereas, at a higher dosage, the toxicity of the toxicological reactions was identical throughout all animal groups.

According to the results of comparative acute toxicity research (intravenous singular dosage) conducted in mice utilizing IONPs that were either uncoated or coated with various materials, 

Including dextran, polyethylene glycol (PEG), albumin, and polylactic acid (PLA), the IC50 values were found to be 355, 355, 232, and 559 mg/kg, correspondingly. As a consequence of these tests, it seems that PLA coatings are more tolerable for NPs than the other coating substances. Pulmonary toxicity, cardiac black material, and moderate splenomegaly were found during the pathological examination of the deceased mice [149]. There was no information provided in this paper on the various NPs’ particle size and coating degree, and this information is critical to understanding the toxic effects of NPs. It was discovered that the mean lethal dosage (LD50) of the aforementioned NPs was more than 2000 mg/kg following oral administration, indicating that oral consumption is more tolerable than a single intravenous infusion of the same dose. When administered intravenously, anhydroglucose polymer (50–150 nm diameter) was shown to be biocompatible since no clinical symptoms of toxicity were noticed throughout rats or mice following the delivery of the IONPs [150]. Dextran stabilized IONPs (AMI-25, Feridex) showed excellent endurance throughout rats and dogs up to 3000 mol Fe/kg, which is about 150 times greater than the dosage necessary for diagnostic hepatic MRI [142]. This suggests that these NPs have a wide security window, which is consistent with previous studies.

**Figure 7:**
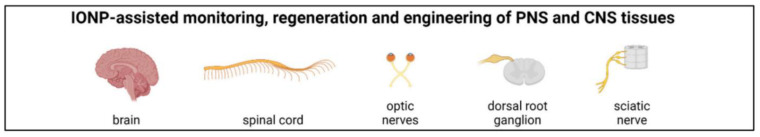
Probable targets for iron oxide nanoparticles-assisted tissue engineering and regenerative tissue engineering for both peripheral and central nervous system [157].

Intravenous injections of IONPs (100-nm-sized nanoparticles covered by anhydroglucose polymer) throughout human tumor individuals were proven to be safe and effective, also IONPs can be applied efficiently for regenerative tissue engineering (Figure 7). NPs administered using doxorubicin (Dox) (overall iron infused per therapeutic session, 132 to 468 mg) were used to treat cancer. The serum iron concentrations were temporarily elevated; however, no hazardous manifestations were noticed, and the quantities of iron in the urine were confirmed to be within the acceptable limit. Toxicological events were not seen in this clinical study, where a magnetic field was given to the upper layer of the skin, and only a small cutaneous discoloration was noticed [151,152]. On the other hand, intravenously administered Ferucarbotran (between 5- and 40-mM Fe/kg) was shown to be safe, except for a transitory reduction in platelet clotting factor XI function, which did not result in any medically meaningful complications. It was also discovered that there were no alterations in cardiac parameters such as blood pressure and cardiac pulse [153,154,155,156,157]. Several mild complications involving the central nervous system (CNS), such as diminished or enhanced voluntary locomotion, rearing, exophthalmos, or mydriasis, have been identified in another research investigation [158]. Human chronic iron toxicity (cirrhosis or hepatocellular cancer) has been reported throughout the publications when large dosages of iron have been administered, with hepatic iron levels above 4 mg Fe/g liver wet weight being seen in certain cases [159].

The connections of NPs with the physiological system and, therefore, their security is influenced by the surface features of NPs, including surficial charge. Nonspecific binding may occur, and cells can be destroyed when neutral or negatively charged NPs bind less effectively with blood constituents and plasma polypeptides than positively charged NPs. The fast elimination from the circulatory system of positively charged NPs also results in nonspecific tissue absorption, as previously stated [160,161].

## 6. Tissue Engineering Applications of Fe_2_O_3_-Based Nanocomposite

**Figure 8:**
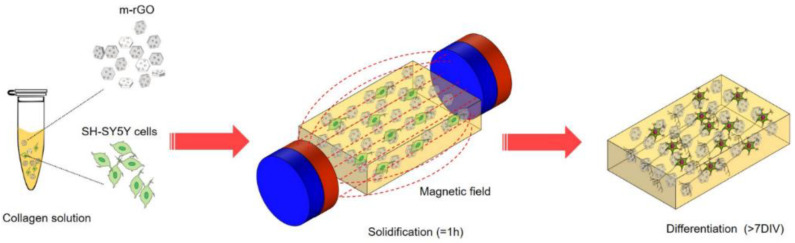
Magnetic-assisted cell alignment for a magnetic-responsive nanocomposite of rGO/collagen hydrogel [168].

Creating substitutes to maintain, restore, or augment the function of injured or damaged tissues is the main goal of tissue engineering [162]. To induce and guide tissue growth, tissue engineering has attempted to utilize scaffolds that mechanically support structure and cells that drive the regeneration process, and bioactive molecules that regulate cell differentiation and functionality [163,164]. Among the polymorphic forms of iron oxides, including γ-Fe_2_O_3_ (maghemite), Fe_3_O_4_ (magnetite), and FeO (wustite), while γ-Fe_2_O_3_ and Fe_3_O_4_ nanoparticles are the most studied ones in tissue engineering due to their exceptional properties, such as paramagnetic, high specific surface area, biocompatibility, nontoxicity, etc., at the nanoscale. Due to these materials’ magnetic response abilities, research in biodegradable magnetic nanocomposites is a rapidly growing topic for biomedical applications (Figure 8), particularly tissue regeneration and musculoskeletal issues such as osteoarthritis [165,166,167,168].

### Fe_2_O_3_-Based Nanocomposites in Bone Tissue Regeneration

The Bone has a special architecture; a more detailed look at the bone structure reveals that it comes into two main parts: compact bone (includes the osteon structures and osteoblast) and spongy bone (the highly porous part) [169]. The bone regeneration procedure begins with the migration and recruitment of osteoprogenitor cells to a flawed section, where they differentiate into osteoblasts and form bone’s ECM mineral part, also known as hydroxyapatite (HA). Magnetic nanoparticles solely or in combination with a magnetic field can help improve and accelerate this process by affecting the three main components of tissue regeneration: (1) Scaffolds (2) Stem cells and (3) Growth factors.

Aside from blood cells, most, if not all, normal cells in human tissues are anchorage-dependent and reside in a solid matrix known as extracellular matrix (ECM). One strategy for bone regeneration is using scaffolds to mimic the natural bone tissue condition. An applicable scaffold must possess a surface that is appropriate for cell attachment, and also should be made with biocompatible substances that act as temporary support to tolerate mechanical forces in the injured zone until the host tissue is completely recuperated. In particular, the biodegradation rate should be matched to the rate of bone formation operation, and the interconnectivity of the porous structure for vascularization is crucial for scaffold design [170].

**Figure 9:**
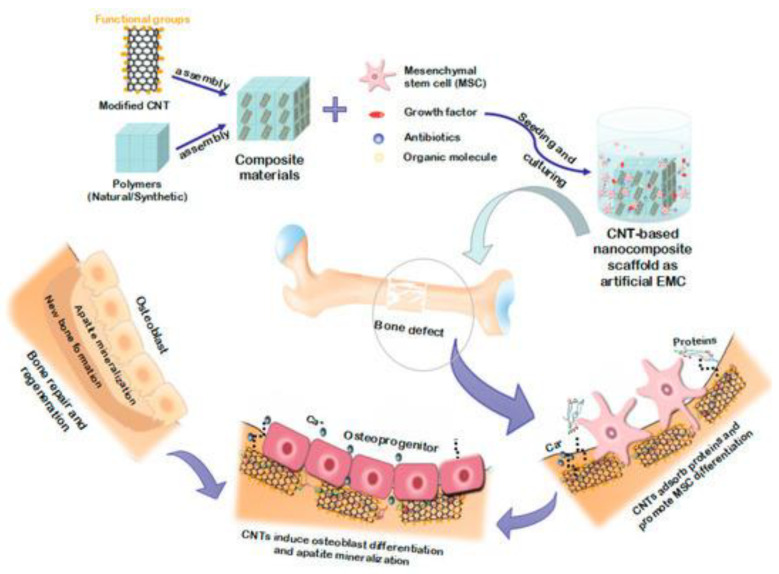
Describes the process of fabricating tissue scaffold and its role in tissue regeneration. Reproduced with permission from [171].

Figure 9 describes how a bone tissue scaffold works in the bone regeneration process. The particular scaffold that has been represented in the Figure 9 is made of carbon nanotube as the base component with combination of polymers. Afterward, mesenchymal stem cell (MSC) with accompanying of appropriate growth factor, antibiotic, and organic molecules was cultured and its effects on bone healing process demonstrated step by step.

The use of magnetic nanoparticles in bone tissue engineering is a growing approach to designing a scaffold. Indeed, magnetic fields have been shown to improve implant integration, and the mineral density of Neotissue, and fasten the defect healing process [172]. These properties associated with the potential of magnetic nanoparticles serving as drug delivery and gene transfection vessels have compelled scientists to conduct extensive research on them in recent years [173]. In a study by Saber-Samandari et al. [174] a combination of these properties was shown. They synthesized bifunctional nanocomposite scaffolds for photothermal therapy and tissue engineering using magnetic nanoparticles as photothermal conversion agents. The incorporation of carboxyl-functionalized multiwalled carbon nanotubes and magnetic iron oxide nanoparticles into the porous matrix of the scaffold caused an increase in BSA (Bovine Serum Albumin) surface adsorption. They also discovered that the presence of magnetic nanoparticles enhanced biocompatibility, as demonstrated by an osteoblastic cell line, as well as the compressive strength of the scaffolds. Świętek et al. [175] created a multifunctional system that allows for multiway cell stimulation by combining hydroxylated MWCNTs with magnetic iron oxide nanoparticles embedded into a porous PCL matrix. Coprecipitation was used to create these hybrid nanoparticles, and the solvent casting/porogen leaching method was used to create the scaffolds. The carbon nanotubes positively affected cell adhesion, according to preliminary research conducted with an osteoblastic cell line. In another study, Hu et al. [176] used poly (lactic-co-glycolic acid)/polycaprolactone/beta-tricalcium phosphate (PPT) scaffolds in another study with γ-Fe_2_O_3_ encapsulation. The γ-Fe_2_O_3_ scaffolds were coated with polyglucose sorbitol carboxymethyl ether. PPT–Fe scaffolds exhibited homogeneous iron distribution and long-term iron release while encapsulated within resorbable PPT scaffolds. Wettability, superparamagnetic, hardness, tensile strength, and elasticity modulus were also observed in that scaffold. Experiments with rat adipose-derived mesenchymal stem cells (rADSCs) in scaffolds revealed an increase in the expression of integrin 1, alkaline phosphatase, and osteogenesis-related genes. Furthermore, improved in vivo bone regeneration was observed after implanting PPT–Fe scaffolds in rat calvarial bone defects. In addition, Meng et al. [177] also developed a novel magnetic nanofibrous composite scaffold by combining γ-Fe_2_O_3_ nanoparticles, hydroxyapatite nanoparticles, and polymer (lactide acid). Under the influence of a static magnetic field, this magnetic scaffold accelerated the formation of new bone tissue in a rabbit model.

A growing number of studies have revealed that IONPs influence BMSC differentiation. On the one hand, the magnetic scaffold itself can induce osteogenesis, as evidenced by data that polymer/IONP (iron oxide nanoparticles) scaffolds can enhance bone regeneration in the presence and absence of a constant magnetic field (CMF) [178,179,180,181]. Cells in interaction with magnetic scaffolds can experience nanoscale stresses analogous to mechanical forces, stretching cell membranes and activating channels and receptors [182]. Furthermore, under cytoplasmic neutral circumstances, IONPs display catalase-like activity, breaking H_2_O_2_ to produce H_2_O with no cytotoxicity [183]. This distinct feature of IONPs increases cell proliferation and accelerates cell cycle progression in MSCs by stimulating the p38 mitogen-activated protein kinase (MAPK) signaling pathway [184,185]. In the acidic environment of endosomes or lysosomes, IONPs break down into Fe^3+^ and PSC molecules. Since these two components will not increase MSC development into osteoblasts, the promotion effect needs the presence of integral IONPs. The studies conducted in this regard are not limited to BMSC differentiation. For instance, Ishmukhametov et al. studied differentiation of adipose-derived MSCs (hMSCs) into chondrocytes, adipocytes and osteoblasts on DNA/Magnetic nanocomposites [186].

Magnetic fields, on the other hand, may be capable of stimulating membrane phospholipid rearrangement and activating the material sensing system of cells, for instance, by activating Ca^2+^ channels on the cell membrane and stimulating various signaling pathways such as the BMP-2, integrin, and MAPK pathways [185].

Kim et al. [187] investigated the effects of electromagnetic fields (45 Hz, 1 mT intensity) and MNPs (50 mg/mL Fe_3_O_4_) on hBMSCs alone or in combination and discovered that both electromagnetic fields and MNPs can enhance osteogenic differentiation, but that using MNPs in combination with electromagnetic field exposure is more operative for enhancing osteogenic differentiation.

In addition, a CMF can impact the shape and orientation of ECM proteins. It has been reported that a unique protein complex (magnetoreceptor, MagR) with an inherent magnetic moment may detect the direction of the magnetic field and deviate from it. MagR is made up of two proteins: iron-sulfur cluster assembly protein (ISCA1) and cryptochrome (human homologous gene Cry1) [188]. According to research, the ISCA1 protein alone is insufficient to cause the cell to react to a magnetic field, hence the protein complex’s stability is critical [189]. The actin cytoskeleton, focal adhesions, integrin binding, stress fibers, and cell responses to mechanical stimulation are all associated with magnetically sensitive proteins. As the MagR protein compound in the cell lines up in the direction of a magnetic field, torque or pressure produce toward the mechanical force receptors such as actin fibers, adhesion sites, and integrins that go along with actin redispersion or integrin-mediated MAPK signaling pathway activation. Thereby, mechanical signals can be transferred to the nucleus to influence the expression of osteogenic genes [190].

The findings of Wang et al. [191] indicated that magnetic IONPs encouraged osteogenic differentiation of MSCs as whole particles and that the IONP-driven magneto genetic response resulted in gene deregulation in their experimental system. The lncRNA INZEB2 is important for osteogenic differentiation because it controls ZEB2 expression as well as the BMP/Smads pathway. Their findings showed how IONPs influence MSC osteogenic differentiation and how lncRNAs contribute to the regulation of magnetic IONP cellular effects.

## 7. Wound Healing Applications of Fe_2_O_3_-Based Nanocomposite

Wound healing is a highly systematic procedure of repairing defected tissue, which is consisted of four consecutive, but overlapping, biological phases: hemostasis, inflammation, proliferation, and remodeling [192,193]. Any disruption by both extrinsic and intrinsic factors in any of the aforementioned phases may cause each phase to be prolonged and result in a dissatisfying result, leading to chronic wound status. The colonization of contaminating pathogens at the spot of damage during the natural wound-healing process is the most commonly occurring issue with complete wound healing [194]. At the spot of a wound, the bacteria that constituted the skin microbiota protect against pathogen colonization. When pathogenic bacteria hit the critical level and generate a large amount of biofilm, the healing process slowed. *Staphylococcus aureus* is the most widely recognized colonizing pathogen influencing the early stages of wound healing, while *Pseudomonas aeruginosa* and *Escherichia coli* are commonly seen in chronic wounds and impact downward layers of the skin [195]. This pathogen-associated bare skin infection may cause acute inflammatory reactions and delayed wound healing. To prevent bacterial infections and assist in the natural wound-healing process, a suitable antimicrobial wound dressing must be used.

However, the proliferation of antimicrobial-resistant bacteria slows the healing process, entailing the development of new wound-dressing substances that are non-toxic or resistant to employment and ameliorating the efficiency of the wound-healing procedure.

In other words, the primary goal of wound dressing is to keep the wound free of extrinsic contamination. It also keeps the wound hydrated, promoting regeneration and preventing the wound’s origin from being exposed [196]. As a result, wound-healing materials must be biocompatible, semi-permeable to water and oxygen, hypoallergenic, and economical. As a result, wound dressing necessitates the use of technologically progressed dressing substances instead of traditional wound-dressing materials such as cotton and wool.

The new materials can preserve the wound environment and also transfer active compounds to facilitate the wound-healing process [197].

Diverse wound-dressing products, including ointments, hydrogels, and antibacterial agents combined with polymers, are recently accessible and are made primarily of biodegradable substances including chitosan, hyaluronic acid, collagen, silicon, cellulose, and gelatin [198,199,200,201,202,203].

Due to their capability to prevent bacterial proliferation, quinolones [204], cephalosporins [205], polymyxin B [206], neomycin [206], and tetracyclines [207] are the most typically utilized antibiotics in wound dressing.

However, repeated and ineffective antibiotic administration would hasten the emergence of antibacterial resistance. To prevent such resistance, treatments based on alternative antibiotics, non-antibiotic materials, and a combination of antibiotics and non-antibiotic materials such as essential oils, honey, and nanomaterials (e.g., Ag and Au) have been recommended for wound dressings [207].

**Figure 10:**
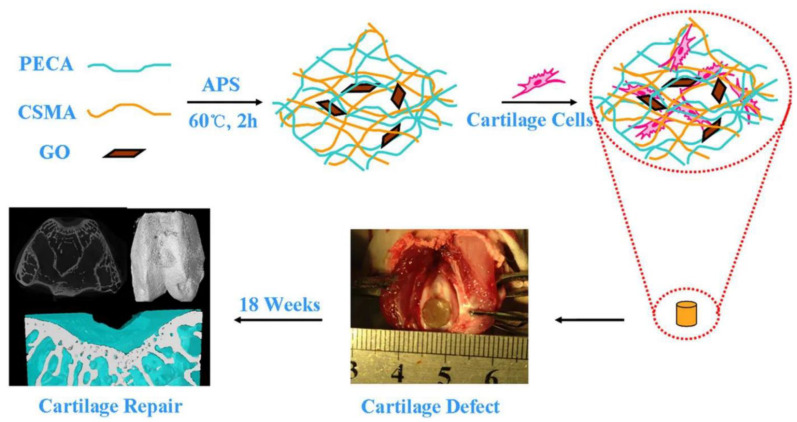
CSMA/PECA/GO hybrid scaffold in bone tissue engineering application (cartilage repairing) [210].

The usage of nanomaterials in wound healing is growing quickly and is being studied in clinical trials [192,208,209,210] (Figure 10). The primary reason for this increase is their physiochemical characteristics, which include nano size, and extended surface with a high specific area. Furthermore, because nanomaterials play a role in active drug delivery, penetrability, and cellular responses, their size and shape are favorable to their use in wound healing [197] In wound therapy, two types of nanomaterials are widely being used (Table 2): (1) nanomaterials with inherent characteristics that typically boost wound treatment, and (2) nanomaterials as carriers for drug delivery.

**Table 2:** The scaffolds fabrication methodology, their characteristics and the effect of the presence of IONPs on their properties, and potential clinical translations have been discussed.

Scaffold Type	Scaffold Fabrication Method	Effects of IONPs on the Scaffold	Ref.
Magnetic hydrogels	nanohydroxyapatite-coated γ-Fe_2_O_3_(around 10 wt%)/PVA composite hydrogels	remarkable influence on the porous structures average pore diameter of: 1.6 ± 0.3 μm enhancing compressive strength: 29.6 ± 6.5 MPa positive impact on osteoblasts adhesion and proliferation	[211]
Magnetic hydrogels	hyaluronic acid/chondroitin sulfate/Fe_2_O_3_/nHAP/PVA hydrogels	Promotion of chondrocyte adhesion, proliferation, and growth	[212]
Electrospinning	electrospun PCL incorporated by dendrimerized superparamagnetic nanoparticles	Significantly decreases the PCL nanofibers size to 495 ± 144 nm and improves cell attachment and growth	[213]
Electrospinning	γ-Fe_2_O_3_ nanoparticles filled polyvinyl alcohol	higher fiber diameter and surface roughness higher cells proliferation rate	[214]
Electrospinning	A novel nanofibrous composite scaffold composed of super-paramagnetic γ-Fe_2_O_3_ nanoparticles (MNP), hydroxyapatite nanoparticles (nHA) and poly lactide acid (PLA)	MNPs accelerates new bone tissue formation and remodeling in the rabbit defect.	[177]
Electrospinning	poly(vinyl alcohol) filled by γ-Fe_2_O_3_ nanoparticles	maximum Young’s modulus (273.51 MPa) cell viability and cell growth rate	[215]
Magnetic Hydrogel	Poly(vinyl alcohol)/nano-hydroxyapatite (n-HA)/magnetic nanoparticles (Fe_2_O_3_) fibers	Enhancing scaffold’s mechanical properties Uniform and enhanced growth of BMSCs on the surface High rates of proliferation Significant simulated chondrocyte-related gene expression	[216]

Due to their inherent nature, metal and metal oxide nanomaterials are more effective in the wound-healing treatment and antibacterial action than traditional materials. Metallic nanoparticles’ size, structure, surface modification, zeta potential, porosity, and thermal stability typically influence their efficacy in biological applications [217,218,219,220]. Considering their favorable physiochemical properties and antibacterial activity, Silver (Ag) [83], gold (Au) [84], and zinc oxide (ZnO) [85] are the most investigated metallic nanoparticles. Other metals and metal oxides, such as iron oxide (Fe_2_O_3_), are also being studied in this regard [195].

Harandi et al. [45] studied the antimicrobial activity of Fe_2_O_3_ nanoparticles as coating for polyvinyl alcohol-prebiotic gum arabic-polycaprolactone (PVA-GA-PCL) electrospun nanofibers for wound dressing applications. Their findings revealed spherical Fe_2_O_3_ NPs with a mean size diameter of 100 nm that contained LAB and demonstrated significant antimicrobial potential against pathogens such as E. coli, Staphylococcus aureus, Pseudomonas aeruginosa, and Candida albicans. These results demonstrated both the biocompatibility of coated nanofibers with mouse embryonic fibroblast cell lines and their antimicrobial and antibiofilm efficacy against pathogens. C. Albicans exhibited the greatest growth inhibition by Fe_2_O_3_ NPs-LAB@PVA-GA-PCL. Intriguingly, combining probiotic Lactobacillus and prebiotic GA as a symbiotic hybrid revealed synergistic antimicrobial effects, implying the therapeutic potential for wound healing applications.

Another study by Raisi et al. [46] fabricated a nanocomposite dressing (NCD) made up of carboxymethyl chitosan (CMC) with various content of Fe2O3 nanoparticles (0, 2.5, 5, and 7.5 wt%) by a method called freeze-drying (FD) technique to study its potential for wound healing applications. The results showed that the wound dress was porous with micron-sized interconnections. Indeed, the results show that as the amount of Fe2O3 nanoparticles increases, so does the porosity. Tensile strength was 0.32 MPa for the pure sample and 0.85 MPa for the sample with the highest percentage of magnetic nanoparticles, respectively.

Furthermore, the cytotoxicity of this nanocomposite revealed no cytotoxicity toward fibroblast cell growth and suitable biocompatibility. The results indicated that NCD had exceptional biodegradability, biocompatibility, and mechanical properties. As a result, NCD made of CMC and Fe_2_O_3_ nanoparticles were proposed as a promising candidate for wound healing applications.

The positive effects of Fe_2_O_3_ NPs on the rapid blood coagulation in wounds with excessive bleeding were proved by a study by Rubtsov et al. [221] in 2019. They synthesized a novel nanoparticle-hydrogel composite as a potential hemostatic wound-healing agent composed of Poly (sodium acrylate) cross-linked by aluminum ions and nanoscale boehmite (γ-AlOOH) and Fe_2_O_3_ as fillers.

## 8. Conclusions

In advanced biomedical systems, scientists are looking for some special features such as good mechanical and thermal stability, biocompatibility, porosity, and biodegradability. These specific characteristics have been fulfilled by employing Fe_2_O_3_ in nanocomposite structures. Thus, IONPs increased drug loading capacity in novel drug delivery and efficiency of tissue engineering applications due to their extended surface area and porosity. In addition, in wound dressings, IONPs have enhanced the permeability of water and oxygen in a more controllable manner.

Due to their incredible superparamagnetic properties, IONPs have been used widely in biomedical engineering applications, especially novel targeted drug delivery systems. This review extensively was devoted to the structural characteristics, and potential applications of IONPs in the areas such as drug delivery, tissue engineering, and wound healing. Different methods of utilizing IONPs in each of those areas have also been discussed and supplied outcomes from recent studies on Fe_2_O_3_-based nanocomposites revealed the applicability of this metal oxide for advanced biomedical purposes.

## Figures and Tables

**Table 1:** Some few biological uses of the newly developed magnetic-responsive devices containing Fe_2_O_3_.

Nanoparticles	Usage	Target	Ref.
Fe_2_O_3_	Magnetic hyperthermia	human hepatocarcinoma SMMC-7721 cells in vitro and xenograft liver cancer in nude mice	[109]
γ-Fe_2_O_3_ NPs (NPs) embedded in a nanohydroxyapatite matrix	Magnetic hyperthermia	Human (Sarcoma osteogenic) SAOS-2 line-cells	[110]
Phosphatidylcholine coated γ-Fe_2_O_3_	Magnetically-induced cell mobility	colon cell lines	[114]
a- Fe_2_O_3_	Drug carrier	human lung fibroblasts (MRC5) cell line	[115]
SiO2/γ-Fe_2_O_3_	Magnetic hyperthermia	BRL-3A cells	[116]
γ-Fe_2_O_3_ nanosheets surface-modified containing polyethylene glycol	Ibuprofen delivery		[117]
polyethyleneimine coated with iron oxide	gene delivery		[113]
hyaluronic acid– Fe_2_O_3_	Peptides delivery		[118]

## Data Availability

Data sharing is not applicable as no new data was created or analyzed in this study.
